# The genitofemoral nerve block: *a* method for hemiscrotal anaesthesia at the bedside

**DOI:** 10.1308/003588412X13171221591259a

**Published:** 2012-05

**Authors:** M Pankhania, S All

**Affiliations:** Milton Keynes General Hospital, Milton KeynesUK

## BACKGROUND

Sensory innervation to the scrotum arises from the genital branch of the genitofemoral nerve, travelling with the spermatic cord through the Inguinal canal en route to the scrotum. It lies immediately lateral to the spermatic cord as it emerges from the superficial Inguinal ring and Is involved in the efferent arm of the cremasteric reflex. This causes distortion and apparent shrinkage of the scrotal surface area, and ascent of the Ipsilateral testis. Many surgeons use local infiltration rather than regional anaesthesia. The scrotal anatomy makes it favourable for regional local anaesthetic nerve blocks to be used when repairing or Incising the scrotal skin.

## TECHNIQUE

The spermatic cord Is identified immediately lateral to the pubic tubercle. The area for injection, including the scrotum, Is sterilised. The spermatic cord is then stabilised and medlallsed using the nondominant hand, and 5ml of 1% lldocaine Is injected subdermally, Immediately lateral to the cord, superficial to the bone.^1^ Negative aspiration prior to injection ensures non-penetrance of the peritoneum or femoral vessels.

## DISCUSSION

The genitofemoral nerve block provides hemiscrotal anaesthesia, allowing painless manipulation and Intervention In an area that is prone to changes In texture and superficial skin anatomy. This method of regional anaesthesia thereby eliminates problems with handling scrotal skin during the time of anaesthetic infiltration, which may occur on stimulation of the cremasteric reflex. This method also minimises the risk of injury to the male genitalia.
